# Supplementary material for: Offspring born to influenza A virus infected pregnant mice have increased susceptibility to viral and bacterial infections in early life
Source: Nat Commun. 2021 Aug 16;12:4957. doi: 10.1038/s41467-021-25220-3 (PMC8368105; doi:10.1038/s41467-021-25220-3)
Supplement: Supplementary file 1 — Supplementary Information [file 41467_2021_25220_MOESM1_ESM.pdf]

Supplementary Information

Offspring born to influenza A virus infected pregnant mice show increased susceptibility to viral and bacterial infections in early life

Henning Jacobsen, Kerstin Walendy-Gnirß, Nilgün Tekin-Bubenheim, Nancy Mounogou Kouassi, Isabel Ben-Batalla, Nikolaus Berenbrok, Martin Wolff, Vinicius Pinho dos Reis, Martin Zickler, Lucas Scholl, Annette Gries, Hanna Jania, Andreas Kloetgen, Arne Düsedau, Gundula Pilnitz-Stolze, Aicha Jeridi, Ali Önder Yildirim, Helmut Fuchs, Valerie Gailus-Durner, Claudia Stoeger, Martin Hrabe de Angelis,Tatjana Manuylova, Karin Klingel, Fiona J Culley, Jochen Behrends, Sonja Loges, Bianca Schneider, Susanne Krauss-Etschmann, Peter Openshaw and Gülsah Gabriel

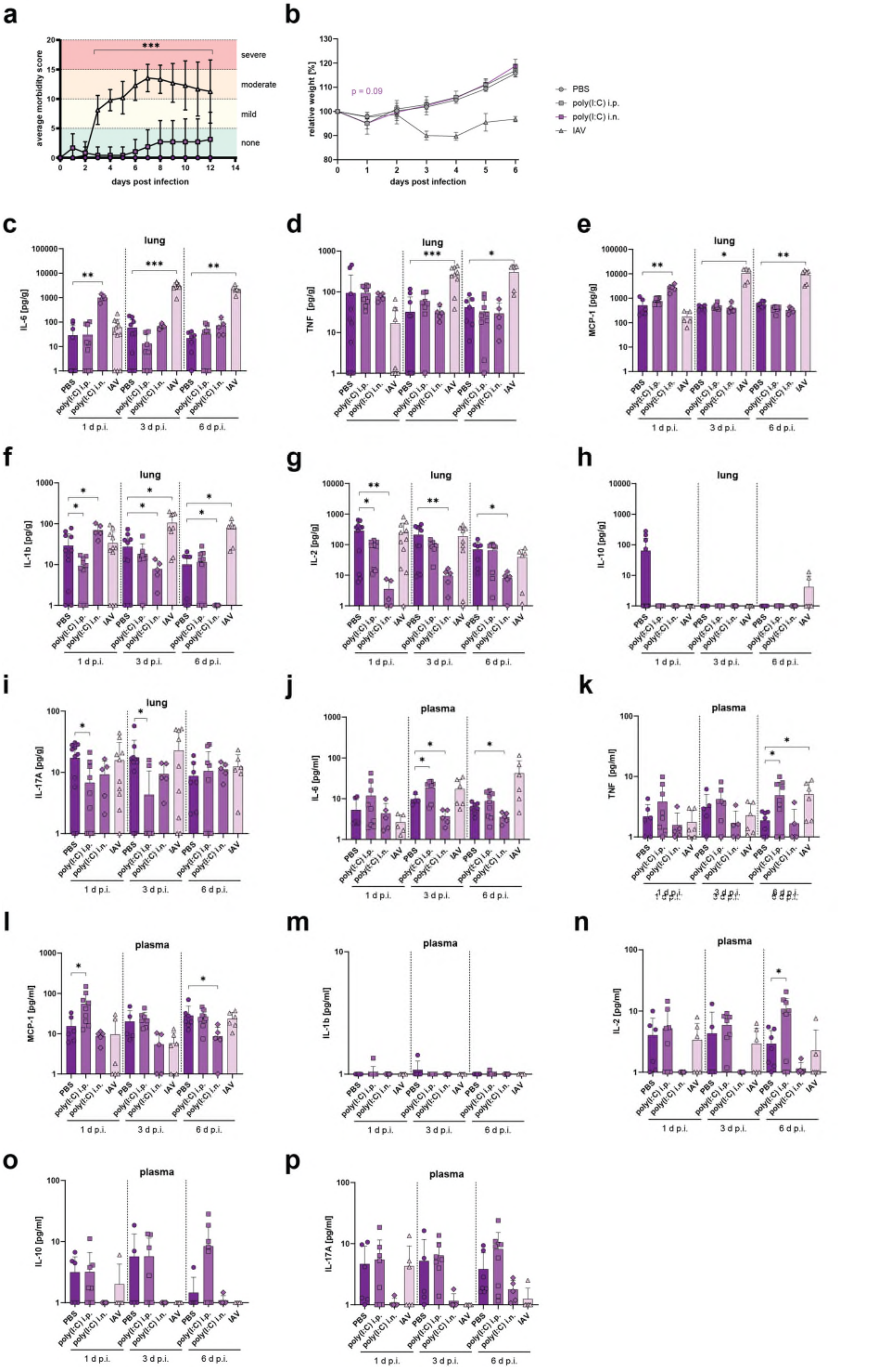

a
average morbidity score
severe
moderate
mild
none
days post infection
b
relative weight [%]
p = 0.09
days post infection
PBS
poly(I:C) i.p.
poly(I:C) i.n.
IAV
c
lung
IL-6 [pg/g]
d
lung
TNF [pg/g]
e
lung
MCP-1 [pg/g]
f
lung
IL-1b [pg/g]
g
lung
IL-2 [pg/g]
h
lung
IL-10 [pg/g]
i
lung
IL-17A [pg/g]
j
plasma
IL-6 [pg/ml]
k
plasma
TNF [pg/ml]
l
plasma
MCP-1 [pg/ml]
m
plasma
IL-1b [pg/ml]
n
plasma
IL-2 [pg/ml]
o
plasma
IL-10 [pg/ml]
p
plasma
IL-17A [pg/ml]
1 d p.i.
3 d p.i.
6 d p.i.

Supplementary Figure 1. 1st hit: poly(I:C)-treatment versus influenza A virus infection. (a) Cumulative morbidity scores (for criteria see Material and Methods) in pregnant mice infected with IAV as shown in Figure 1 (triangles, n = 24), intraperitoneal (i.p.) poly(I:C) (rectangles, n = 24); p<0.0001. (b) Weight of pregnant C57BL/6 mice after treatment with intranasal (i.n.) phosphate-buffered saline (PBS) (n = 9), i.p. poly(I:C) (n = 8), i.n. poly(I:C) (n = 15; p=0.0880) or i.n. IAV (n = 7) until 6 days post infection (d p.i.) (c-i) Cytokines (IL-6; p=0.0019 (1 d p.i.), p<0.0001 (3 d p.i.), p=0.0010 (6 d p.i.) [c], TNF; p=0.0010 (3 d p.i.), p=0.0103 (6 d p.i.) [d], MCP-1; p=0.0027 (1 d p.i.), p=0.0195 (3 d p.i.), p=0.0058 (6 d p.i.) [e], IL-1β; p=0.0322 (poly(I:C) i.p. 1 d p.i.), p=0.0200 (poly(I:C) i.n. 1 d p.i.), p=0.0400 (poly(I:C) i.n. 3 d p.i.), p=0.0111 (3 d p.i.), p=0.0309 (poly(I:C) i.n. 6 d p.i.), p=0.0144 (6 d p.i.) [f], IL-2; p=0.0161 (poly(I:C) i.p. 1 d p.i.), p=0.0025 (poly(I:C) i.n. 1 d p.i.), p=0.0073 (poly(I:C) i.n. 3 d p.i.), p=0.0185 (poly(I:C) i.n. 6 d p.i.) [g], IL-10 [h] and IL-17A; p=0.0204 (1 d p.i.), p=0.0321 (3 d p.i.) [i]) determined by Luminex assay in the lungs of pregnant mice treated with PBS i.n. (n = 10 [IL-6, 1 d p.i.], 10 [IL-6, 3 d p.i.], 7 [IL-6, 6 d p.i.], 11 [TNF, 1 d p.i.], 10 [TNF, 3 d p.i.], 7 [TNF, 6 d p.i.], 6 [MCP-1, 1 d p.i.], 5 [MCP-1, 3 d p.i.], 7 [MCP-1, 6 d p.i.]), poly(I:C) i.p. (n = 10 [IL-6, 1 d p.i.], 8 [IL-6, 3 d p.i.], 9 [IL-6, 6 d p.i.], 10 [TNF, 1 d p.i.], 8 [TNF, 3 d p.i.], 9 [TNF, 6 d p.i.], 11 [MCP-1, 1 d p.i.], 9 [MCP-1, 3 d p.i.], 10 [MCP-1, 6 d p.i.]), poly(I:C) i.n. (n = 5 [IL-6, 1 d p.i.], 5 [IL-6, 3 d p.i.], 5 [IL-6, 6 d p.i.], 5 [TNF, 1 d p.i.], 5 [TNF, 3 d p.i.], 5 [TNF, 6 d p.i.], 5 [MCP-1, 1 d p.i.], 5 [MCP-1, 3 d p.i.], 5 [MCP-1, 6 d p.i.]) or infected with IAV i.n. (n = 11 [IL-6, 1 d p.i.], 9 [IL-6, 3 d p.i.], 6 [IL-6, 6 d p.i.], 10 [TNF, 1 d p.i.], 9 [TNF, 3 d p.i.], 6 [TNF, 6 d p.i.], 5 [MCP-1, 1 d p.i.], 5 [MCP-1, 3 d p.i.], 6 [MCP-1, 6 d p.i.]) at E5.5, measured at 1, 3 and 6 d p.i. (j-p) Cytokines (IL-6; p=0.0343 (poly(I:C) i.p. 3 d p.i.), p=0.0151 (poly(I:C) i.n. 3 d p.i.), p=0.0115 (poly(I:C) i.n. 6 d p.i.) [j], TNF; p=0.0218 (poly(I:C) i.p. 6 d p.i.), p=0.0429 (6 d p.i.) [k], MCP-1; p=0.0329 (1 d p.i.), p=0.0385 (6 d p.i.) [l], IL-1β [m], IL-2; p=0.0209 (6 d p.i.) [n], IL-10 [o] and IL-17A [p]) determined by Luminex assay in plasma samples of pregnant mice treated with PBS i.n. (n = 6 [IL-6, 1 d p.i.], 4 [IL-6, 3 d p.i.], 7 [IL-6, 6 d p.i.], 6 [TNF, 1 d p.i.], 5 [TNF, 3 d p.i.], 7 [TNF, 6 d p.i.], 6 [MCP-1, 1 d p.i.], 5 [MCP-1, 3 d p.i.], 7 [MCP-1, 6 d p.i.]), poly(I:C) i.p. (n = 9 [IL-6, 1 d p.i.], 7 [IL-6, 3 d p.i.], 9 [IL-6, 6 d p.i.], 8 [TNF, 1 d p.i.], 7 [TNF, 3 d p.i.], 9 [TNF, 6 d p.i.], 9 [MCP-1, 1 d p.i.], 7 [MCP-1, 3 d p.i.], 9 [MCP-1, 6 d p.i.]), poly(I:C) i.n. (n = 5 [IL-6, 1 d p.i.], 5 [IL-6, 3 d p.i.], 5 [IL-6, 6 d p.i.], 5 [TNF, 1 d p.i.], 5 [TNF, 3 d p.i.], 5 [TNF, 6 d p.i.], 5 [MCP-1, 1 d p.i.], 5 [MCP-1, 3 d p.i.], 5 [MCP-1, 6 d p.i.]) or infected with IAV i.n. (n = 5 [IL-6, 1 d p.i.], 6 [IL-6, 3 d p.i.], 6 [IL-6, 6 d p.i.], 6 [TNF, 1 d p.i.], 6 [TNF, 3 d p.i.], 6 [TNF, 6 d p.i.], 6 [MCP-1, 1 d p.i.], 6 [MCP-1, 3 d p.i.], 6 [MCP-1, 6 d p.i.]) at E5.5, measured at 1, 3 and 6 d p.i. Values are normalized to organ weight if applicable. All data are presented as mean and SD. Different groups are depicted in dark circles (PBS), medium squares (Poly(I:C)) or light triangles (IAV) in violet colors. Cytokine levels that were below detection limit were set to the kit's lower detection limit of 1 pg/g. Data for IL-6, TNF and MCP-1 for the groups PBS i.n., poly(I:C) i.p. and IAV i.n. are also shown in figure 1 and are used as a reference for poly(I:C) i.n. in this figure. The statistical significance was calculated by multiple, two-tailed t-test using the Bonferroni-Dunn correction (a and b), or by two-tailed Welch's t-test (c-p) (*p<0.05, **p<0.01, ***p<0.001). PBS treated groups were used as reference to compare to IAV infected groups in all statistical analyses unless stated otherwise. Non-significant comparison are not depicted within the respective figures. Source data are provided as a Source Data file.

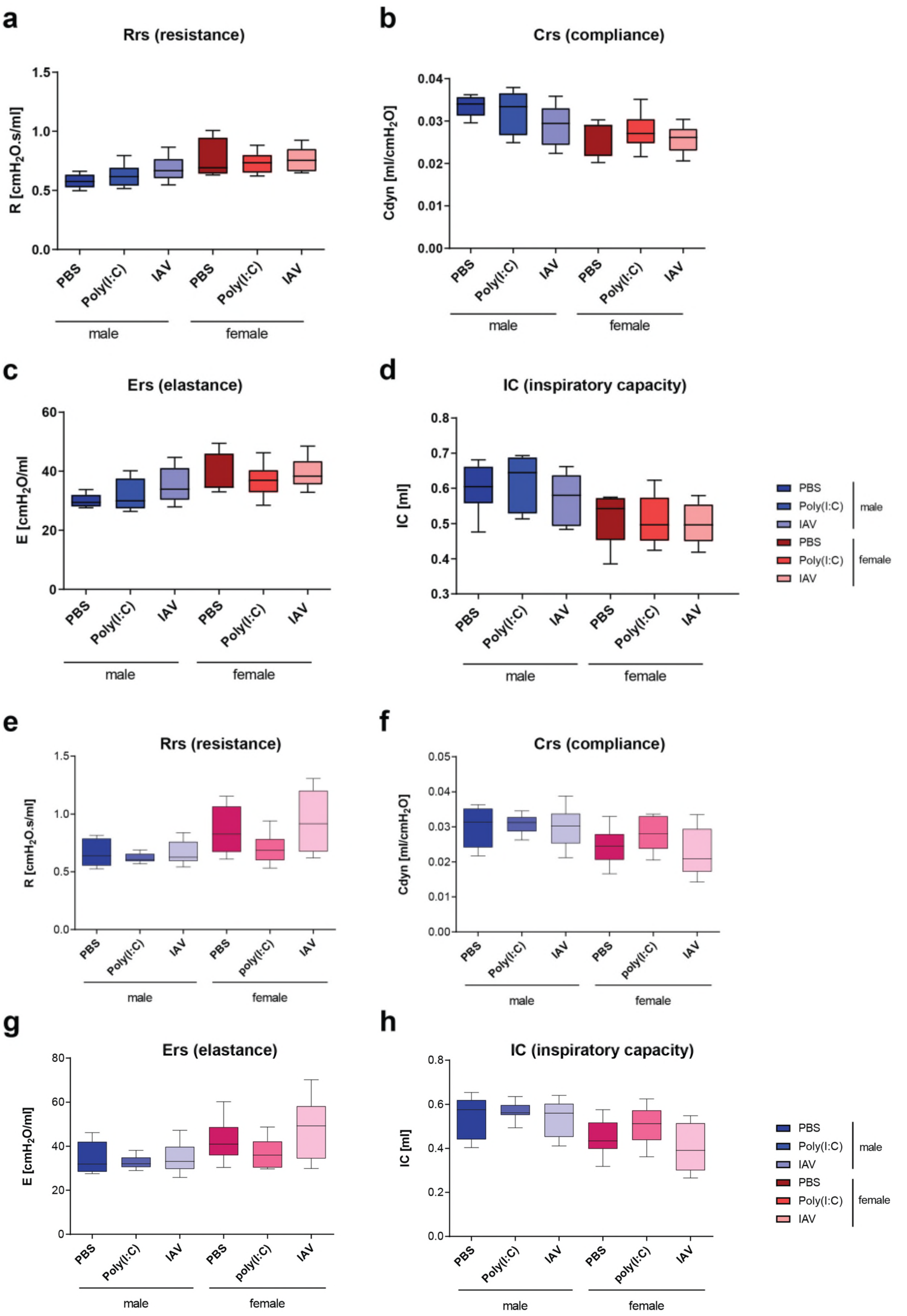

Supplementary Figure 2. Offspring's lung physiology and function. Lung function was assessed in 6-week-old offspring (a) resistance (Rrs measured in R [cmH_2O.s/ml]), (b) compliance (Crs measured in Cdyn [ml/cmH_2O]), (c) elastance (Ers measured in E [cmH_2O/ml]), (d) inspiratory capacity (IC in ml) as well as 20-week-old offspring (n = 8) (e) resistance, (f) compliance, (g) elastance, (h) inspiratory

capacity born to phosphate-buffered saline (PBS)-treated (n = 8 males, n = 7 females), polyinosinic: polycytidylic acid (poly(I:C))-treated (n = 8 males, n = 7 females) or influenza A virus (IAV)-infected (n = 9 males, n = 8 females) dams using a FlexiVent Research System. All data are presented as box plot showing min to max with median and ± SEM. Different groups of male offspring are depicted in dark circles (PBS), medium squares (Poly(I:C)) or light triangles (IAV) in blue colors and female offspring in red colors. Statistical significance was calculated using one-way ANOVA. Non-significant comparison are not depicted within the respective figures. Source data are provided as a Source Data file.

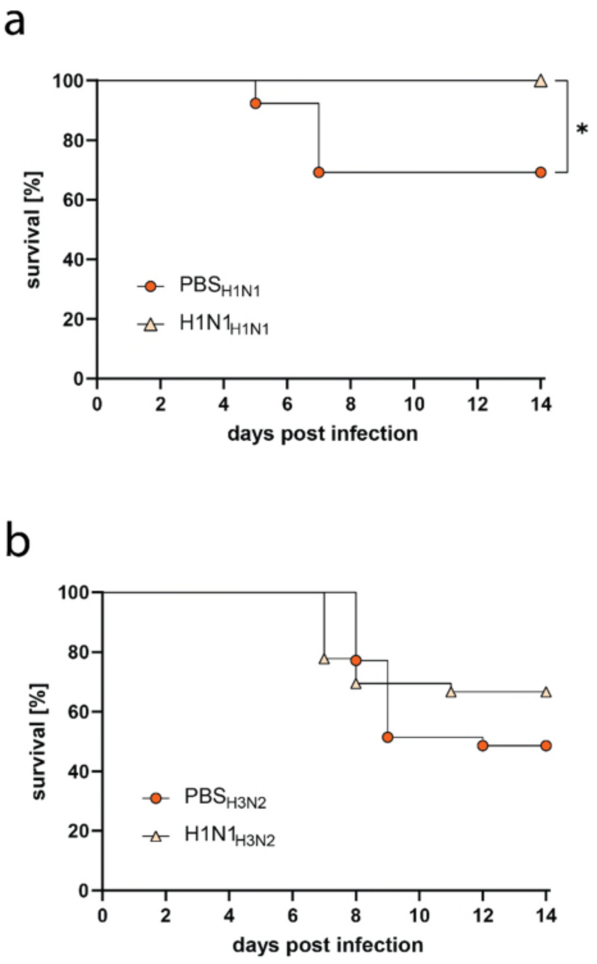

Supplementary Figure 3. Homologous second hit experiments in juvenile offspring. (a) 2-week-old offspring born to phosphate-buffered saline (PBS)- (n = 13) or influenza A virus (IAV)-infected (n = 14) dams infected with 10^3 plaque forming units (PFU) of IAV (H1N1). Survival was determined within 14 days post infection (d p.i.); p=0.0274 (b) 2-week-old offspring born to PBS- (n = 35) or IAV-infected (n = 37) dams infected with 10^2 PFU of IAV (H3N2 6+2 reassortant in WSN). Survival was determined within 14 d p.i. All n represent number of offspring from respective groups. Different groups of offspring without stratification by sex are depicted in dark circles (PBS-treated mothers) or light triangles (IAV-infected mothers) in orange colors. The statistical significance was calculated using Log Rank test (a-b) (*$p<0.05$). PBS treated groups were used as reference to compare to IAV infected groups in all statistical analyses unless stated otherwise. Non-significant comparison are not depicted within the respective figures. Source data are provided as a Source Data file.

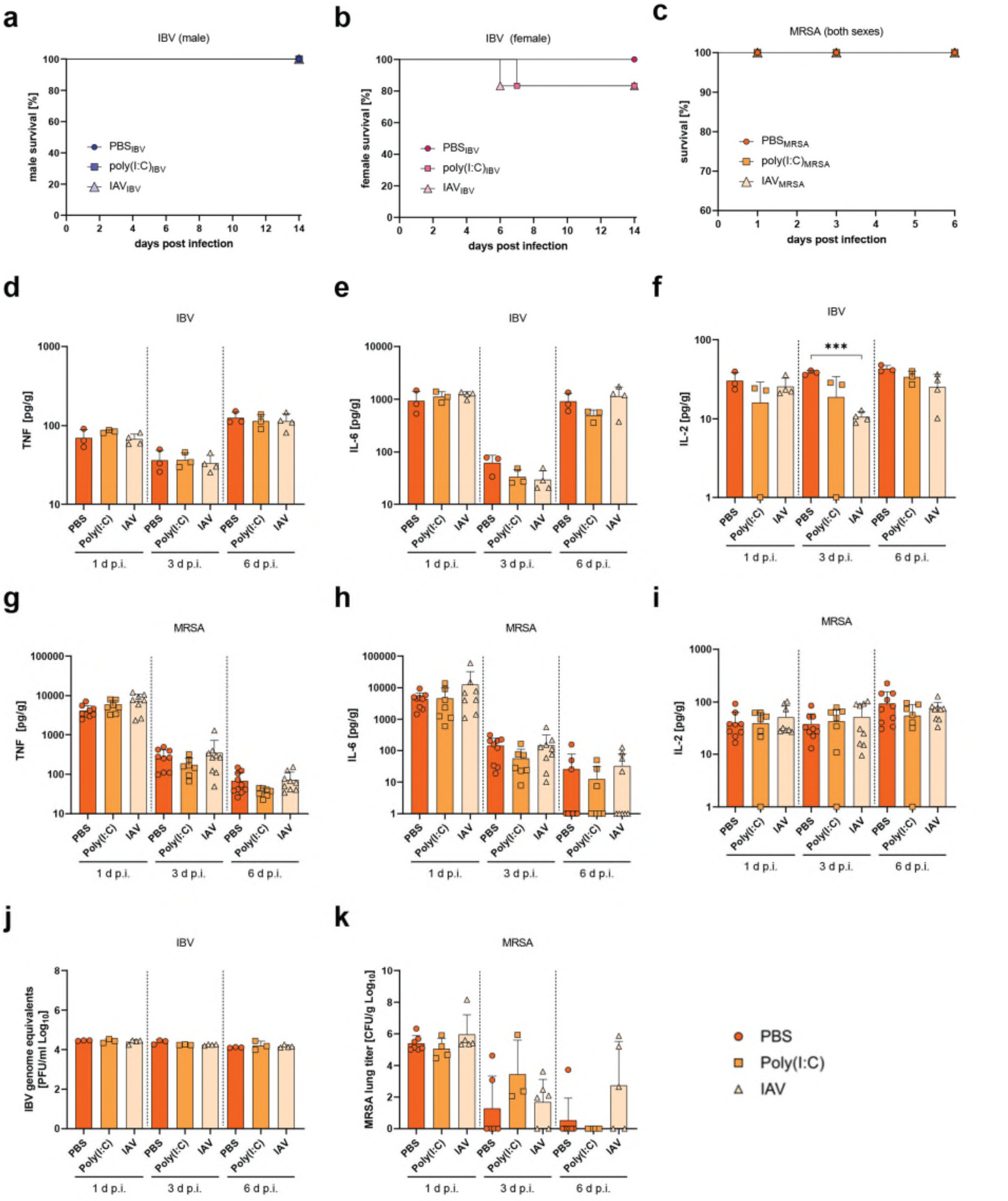

Supplementary Figure 4. Second hit experiments in adult offspring. (a) 6-week-old male offspring born to phosphate-buffered saline (PBS)- (n = 5), polyinosinic:polycytidylic acid (poly(I:C))-treated (n = 8) or influenza A virus (IAV)-infected (n = 8) dams infected with 10^5 plaque forming units (PFU) of influenza B virus (IBV). Survival was determined within 14 days post infection (d p.i.) (b) 6-week-old female offspring born to PBS- (n = 4), poly(I:C)-treated (n = 6) or IAV-infected (n = 6) dams infected with 10^5 PFU of IBV. Survival was determined within 14 d p.i. (c) 6-week-old offspring (both sexes) born to poly(I:C)-treated (n = 21 [day 1], 14 [day 3], 7 [day 6]) or IAV-infected (n = 36 [day 1], 25 [day 3], 13 [day 6]) dams infected with 10^8 colony forming units (CFU) of Methicillin-resistant *Staphylococcus aureus* (MRSA). Offspring born to PBS-treated dams (n = 34 [day 1], 22 [day 3], 11 [day 6]) were used as controls. Survival was determined at day 1, 3 or 6 p.i. (d-f) Cytokines (TNF [d], IL-6 [e], and IL-2, p=0.0002 (3d p.i.) [f]) determined by Luminex assay in lungs of 6-week-old offspring (both sexes) born to PBS- (n = 3) poly(I:C)-treated (n = 3) or IAV-infected offspring (n = 4) infected with 10^5 PFU of IBV

measured at 1, 3 or 6 d p.i. (g-i) Cytokines (TNF [g], IL-6 [h], and IL-2 [i]) determined by Luminex assay in plasma of 6-week-old offspring (both sexes) born to PBS- (n = 9 [1 and 3 d p.i.], 10 [6 d p.i.]), poly(I:C) (n = 7) or IAV-infected offspring (n = 8 [1 and 3 d p.i.], 9 [6 d p.i.]) infected with 10^8 CFU of MRSA measured at 1, 3 or 6 d p.i. (j) IBV lung titer in 6-week-old offspring (both sexes) born to PBS- (n = 3), poly(I:C)-treated (n = 3) or IAV-infected (n = 4) dams, measured on day 1, 3 and 6 p.i. Values are normalized to organ weight. (k) MRSA lung titer in 6-week-old offspring (both sexes) born to PBS- (n = 7 [1 d p.i.], 6 [3 d p.i.], 7 [6 d p.i.]), poly(I:C)-treated - (n = 4 [1 d p.i.], 3 [3 d p.i.], 4 [6 d p.i.]) or IAV-infected - (n = 5 [1 d p.i.], 6 [3 d p.i.], 5 [6 d p.i.]) dams, measured on day 1, 3 and 6 p.i. All n represent number of offspring from respective groups. Data in (d-i) are presented as mean and SD. Different groups of male offspring are depicted in dark circles (PBS), medium squares (Poly(I:C)) or light triangles (IAV) in blue colors and female offspring in red colors. Different groups of offspring that were not stratified by sex are depicted in orange colors with the same icons. Cytokine levels that were below detection limit were set to the kit's lower detection limit of 1 pg/g. The statistical significance was calculated by two-tailed Welch's t test (d-i) or Log Rank test (a-c) (***p<0.001). PBS treated groups were used as reference to compare to IAV infected groups in all statistical analyses unless stated otherwise. Non-significant comparison are not depicted within the respective figures. Source data are provided as a Source Data file.

a

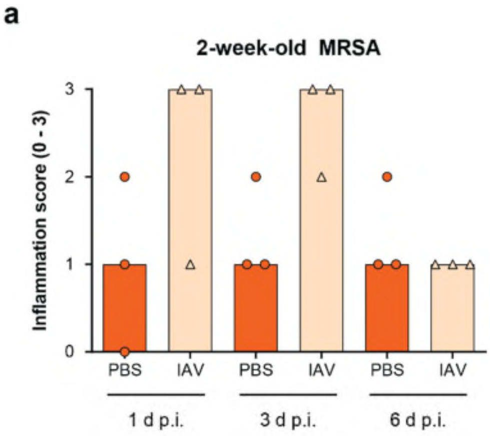

b

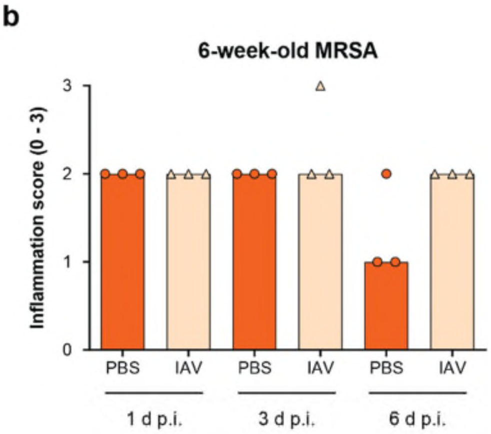

c

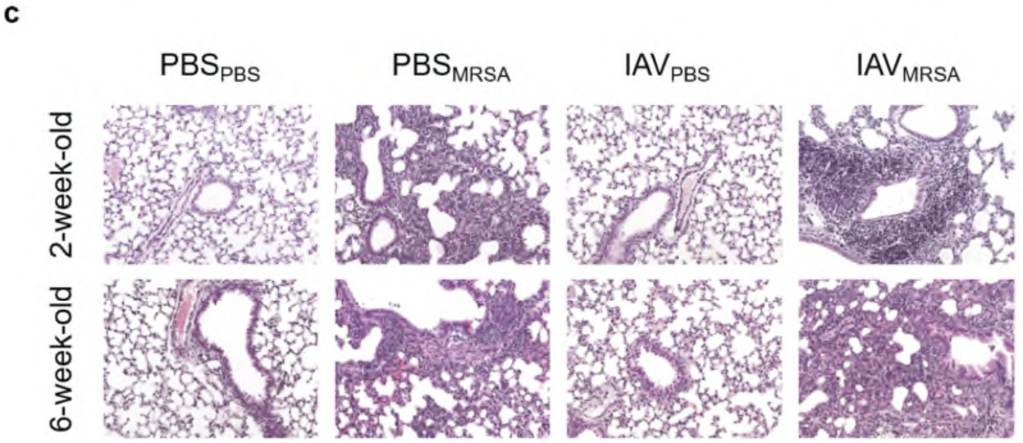

d

		1 d p.i.		3 d p.i.		6 d p.i.	
		Inflammation	Score	Inflammation	Score	Inflammation	Score
2-week-old	PBS	None	0	Granulocytic peri-bronchial and interstitial	2	Slight inflammation	1
		Slight granulocytic peri-bronchial	1	Granulocytic peri-bronchial and interstitial	2	Medium interstitial	2
		Granulocytic peri-bronchial and interstitial	2	Slight granulocytic peri-bronchial	1	Sub-pleural infiltrate	1
	IAV	Slight granulocytic peri-bronchial and interstitial	1	Severe granulocytic peri-bronchial and interstitial	3	Slight granulocytic peri-bronchial	1
		Severe granulocytic peri-bronchial and interstitial	3	Severe granulocytic peri-bronchial and interstitial	3	Slight granulocytic peri-bronchial	1
		Severe granulocytic peri-bronchial and interstitial	3	Severe inflammation	3	Slight granulocytic peri-bronchial	1
6-week-old	PBS	Medium granulocytic peri-bronchial	2	Medium inflammation peri-bronchial and interstitial	2	Medium inflammation interstitial	2
		Medium inflammation peri-bronchial and interstitial	2	Medium inflammation peri-bronchial and interstitial	2	Slight peri-bronchial inflammation	1
		Medium inflammation peri-bronchial and interstitial	2	Medium inflammation interstitial	2	Slight peri-bronchial inflammation	1
	IAV	Medium inflammation peri-bronchial and interstitial	2	Medium peri-bronchial inflammation	2	Medium peri-bronchial inflammation	2
		Medium inflammation interstitial	2	Medium inflammation peri-bronchial and interstitial	2	Medium peri-bronchial inflammation	2
		Medium inflammation peri-bronchial and interstitial	2	Severe granulocytic peri-bronchial and interstitial	3	Medium peri-bronchial inflammation	2

Supplementary Figure 5. Lung histology of offspring after MRSA second hit. (a and b) Inflammation scores based on histological assessment of lungs from two-week-old (a) and six-week-old (b) offspring (n = 3) born to phosphate-buffered saline (PBS)-treated or influenza A virus (IAV)-infected dams after second hit with 10^8 colony forming units (CFU) of methicillin-resistant *Staphylococcus aureus* (MRSA). (c) representative histology of offspring's lungs 3 days post-treatment/infection with Hematoxylin/Eosin (HE) staining. The width of each individual picture corresponds to 550 µm. Per group three animals were assessed as shown in panel d. (d) Histological assessment and assigned score for each lung analyzed. Different groups of offspring are depicted in dark circles (PBS), or light triangles (IAV) in orange colors. Data presented in (a and b) are shown as individual data points with median. Additional representative histology is are provided as a Source Data file.

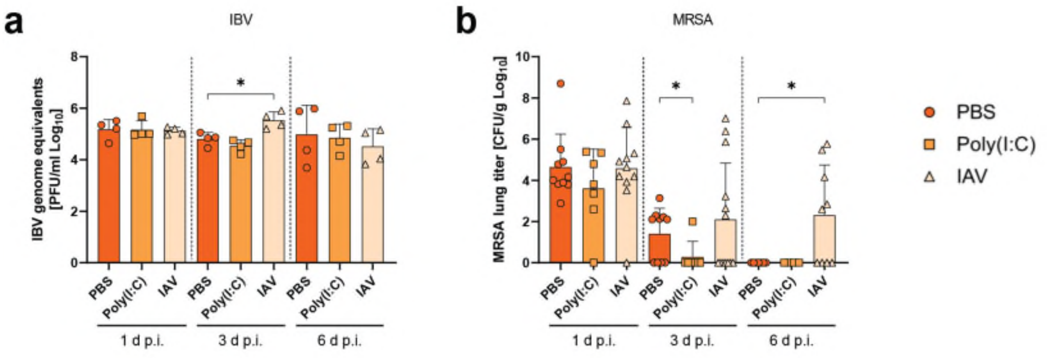

Supplementary Figure 6. Viral and bacterial titers in the lungs of offspring born to dams infected with IAV. (a) Influenza B virus (IBV) lung titers in 2-week-old offspring (both sexes) born to phosphate-buffered saline (PBS)-, polyinosinic:polycytidylic acid (poly(I:C)-treated or influenza A virus (IAV)-infected (n = 4) dams, measured on day 1, 3 and 6 post infection (d.p.i.); p=0.0126. (b) Methicillin-resistant *Staphylococcus aureus* (MRSA) lung titers in 2-week-old offspring (both sexes) born to PBS- (n = 10 [1 d p.i.], 10 [3 d p.i.], 7 [6 d p.i.]), poly(I:C)-treated (n = 7 [1 d p.i.], 7 [3 d p.i.], 4 [6 d p.i.]) or IAV-infected (n = 11 [1 d p.i.], 13 [3 d p.i.], 9 [6 d p.i.]) dams, measured on day 1, 3 and 6 p.i.; p=0.0368 (3d p.i.), p=0.0211 (6d p.i.). Values are normalized to organ weight. All n represent number of male and/or female offspring from respective groups. Groups were merged for clarity if no difference between males and females was observed. Different groups of offspring are depicted in dark circles (PBS), medium squares (Poly(I:C)) or light triangles (IAV) in orange colors. Data are presented as individual values with mean ± SD. The statistical significance was calculated by two-tailed Welch´s t-test. PBS treated groups were used as reference to compare to IAV infected groups in all statistical analyses unless stated otherwise. Source data are provided as a Source Data file.

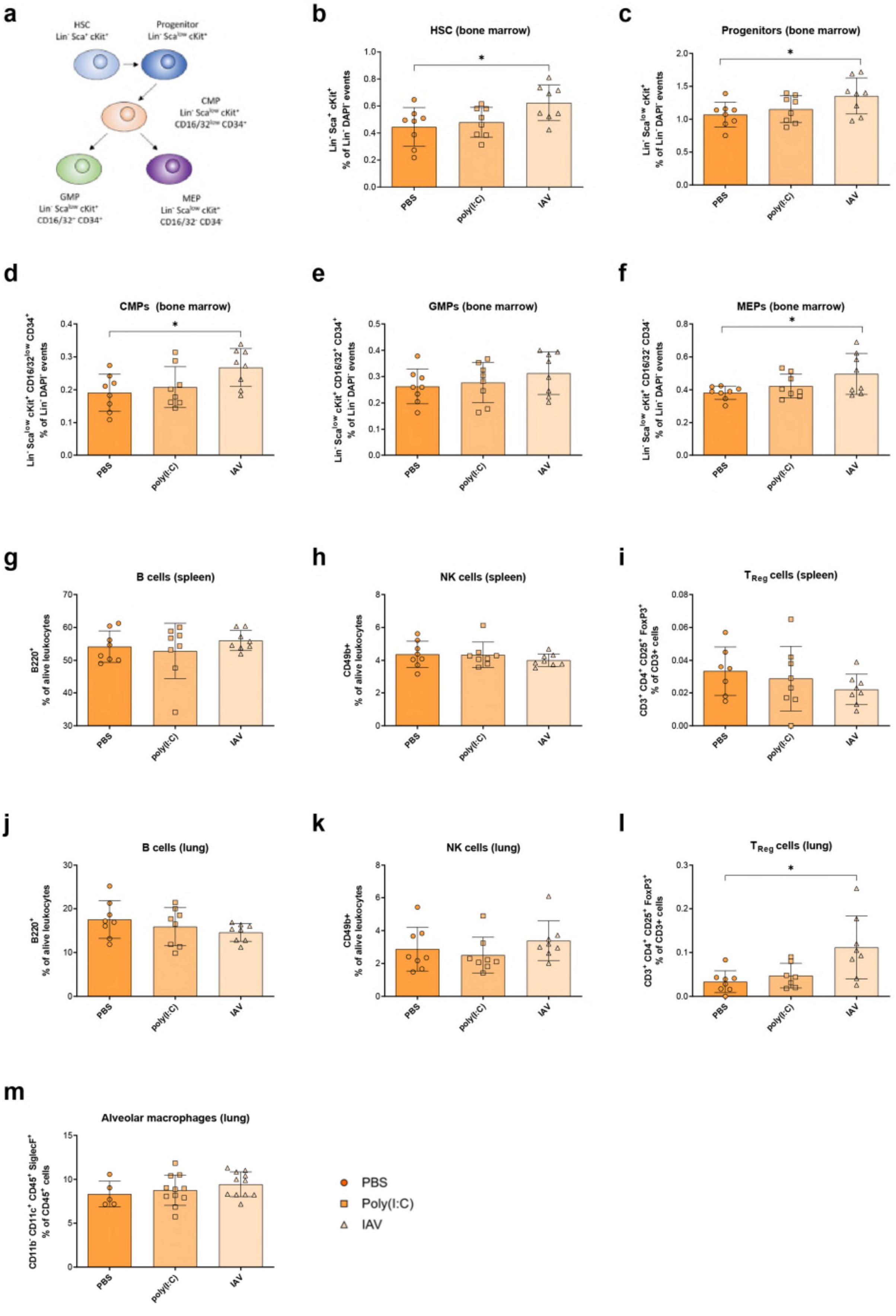

Supplementary Figure 7. Stem- and immune cell frequencies in adult offspring. (a) Markers used to define respective stem (HSC) and progenitor cell populations by flow cytometry in this study. (b-f)

Frequency of Lin^- Sca^+ $cKit^+$ HSC; p=0.0210 (b), Lin^- Sca^{low} $cKit^+$ progenitor cells; p=0.0322 (c), Lin^- Sca^{low} $cKit^+$ $CD16/32^{low}$ $CD34^+$ common myeloid progenitor (CMP) cells; p=0.0176 (d), Lin^- Sca^{low} $cKit^+$ $CD16/32^+$ $CD34^+$ granulocyte-monocyte progenitor (GMP) cells (e) and Lin^- Sca^{low} $cKit^+$ $CD16/32^-$ $CD34^-$ megakaryocyte-erythrocyte progenitor (MEP) cells; p=0.0373 (f) as % of Lin^- $DAPI^-$ events in the bone marrow of 6-week-old offspring (n = 4) born to early gestational polyinosinic:polycytidylic acid (PBS)-treated, polyinosinic:polycytidylic acid (poly(I:C))-treated or influenza A virus (IAV)-infected dams, as assessed by flow cytometry. (g-l) Frequency of $B220^+$ B cells (g and i), $CD49b^+$ natural killer (NK) cells (h and k), $CD3^+$ $CD4^+$ $CD25^+$ $FoxP3^+$ regulatory T (T_{Reg}) cells (i and l; p=0.0178) as % of alive leukocytes in spleens (g-i) or lungs (j-l) of 6-week-old offspring (n = 4) born to early gestational PBS-treated, poly(I:C)-treated or IAV-infected dams, as assessed by flow cytometry. (m) Frequency of $CD11b^-$ $CD11^+$ $CD45^+$ $SiglecF^+$ alveolar macrophages as % of $CD45^+$ cells in lungs of 6-week-old offspring born to early gestational PBS-treated (n = 5), poly(I:C)-treated (n = 11) or IAV-infected dams (n = 11), as assessed by flow cytometry. All n represent number of male and/or female offspring from respective groups. Groups were merged for clarity as no difference between males and females was observed. All data are presented as mean and SD. Different groups of offspring are depicted in dark circles (PBS), medium squares (Poly(I:C)) or light triangles (IAV) in orange colors. The statistical significance was calculated by two-tailed Welch's t (*p<0.05). PBS treated groups were used as reference and compared to IAV infected groups in all statistical analyses unless stated otherwise. Source data are provided as a Source Data file.

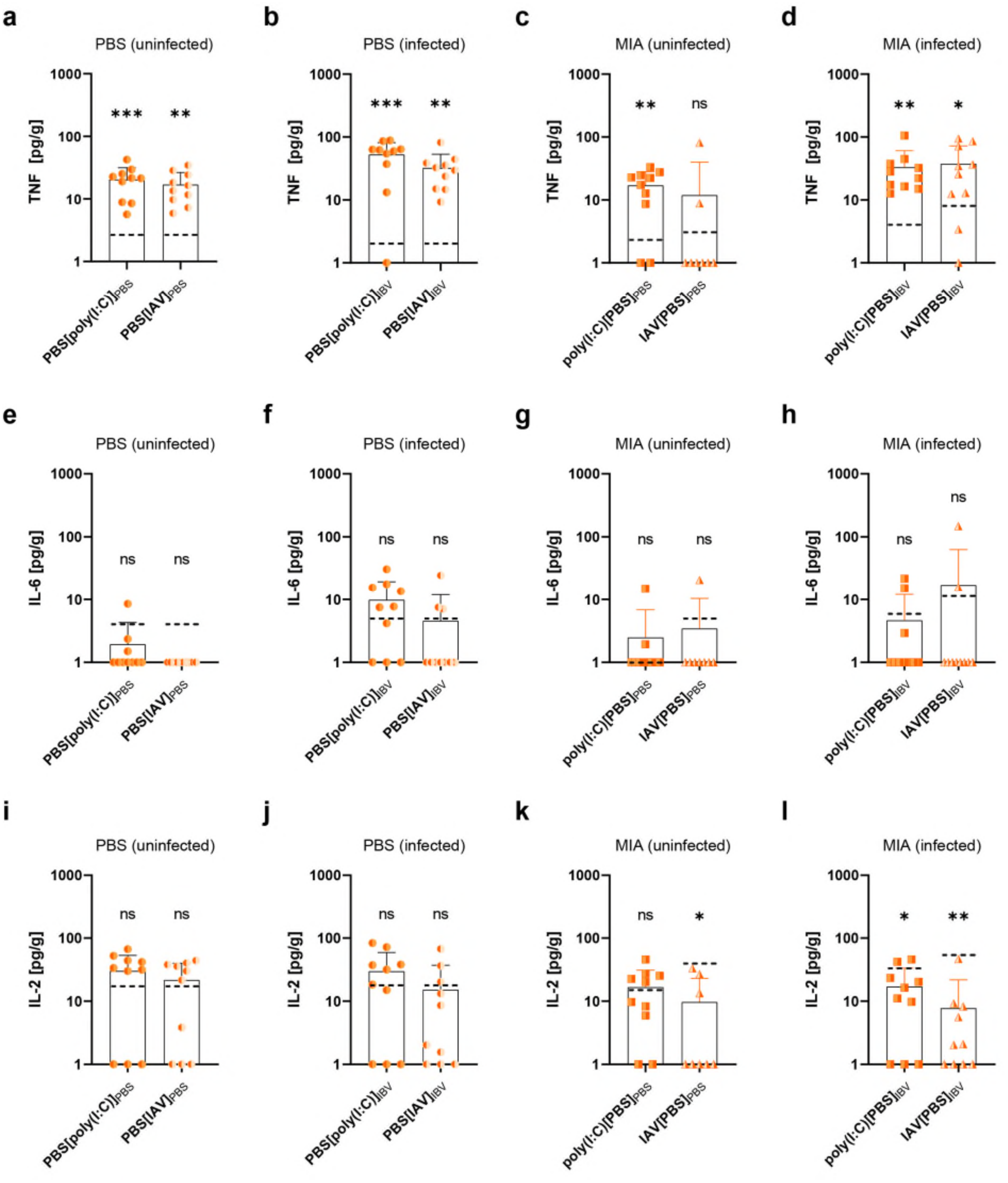

Supplementary Figure 8. Lung cytokine levels in 2-week-old offspring after adoptive transfer of alveolar macrophages. (a and b) TNF levels from mock-infected; p=0.0005 and p=0.0010 (a) or influenza B virus (IBV)-infected; p=0.0003 and p=0.0012 (b) offspring (3 days post infection (d p.i.)) born to phosphate-buffered saline (PBS)-treated dams and after transfer of alveolar macrophages (AMs) from offspring born to polyinosinic:polycytidylic acid (poly(I:C))-treated or influenza A virus (IAV)-infected dams. (c and d) TNF levels from mock-infected; p=0.0017 and not significant (n. s.) (c) or IBV-infected; p=0.0087 and p=0.0312 (d) offspring (3 d p.i.) born to maternal immune activation (MIA)-treated dams and after transfer of AMs from offspring born to PBS-treated dams. (e and f) IL-6 levels from mock-infected (e) or IBV-infected (f) offspring (3 d p.i.) born to PBS-treated dams and after transfer of AMs from offspring born to poly(I:C)-treated or IAV-infected dams. (g and h) IL-6 levels from mock-infected (g) or IBV-infected (h) Offspring (3 d p.i.) born to MIA-treated dams and after transfer of AMs from offspring born to PBS-treated dams. (i and j) IL-2 levels from mock-infected (i) or IBV-infected (j) offspring (3 d p.i.) born to PBS-treated dams and after transfer of AMs from offspring born to poly(I:C)-treated or IAV-infected dams. (k and l) IL-2 levels from mock-infected; n. s. and p=0.0311 (k) or IBV-infected; p=0.0143 and p=0.0066 (l) offspring (3 d p.i.) born to MIA-treated dams and after transfer of

AMs from offspring born to PBS-treated dams. N for all groups is 10 (5 males and 5 females), except for (c), (g) and (k), where n for IAV[PBS]$_{PBS}$ is 8 (3 males, 5 females). Dotted lines indicate reference values for each respective group from mock-infected or IBV-infected offspring without prior AM transfer and were taken from figure 3. Values are normalized to organ weight. All data in are presented as mean and SD. Different groups of offspring are depicted in dark circles (PBS), medium squares (Poly(I:C)) or light triangles (IAV) in orange colors. Cytokine levels that were below detection limit were set to the kit's lower detection limit of 1 pg/g. Statistics were performed for each group and its reference. The statistical significance was calculated by two-tailed Welch's t test (*p<0.05, **p<0.01, ***p<.001). PBS treated groups were used as reference and compared to IAV infected groups in all statistical analyses unless stated otherwise. Source data are provided as a Source Data file.

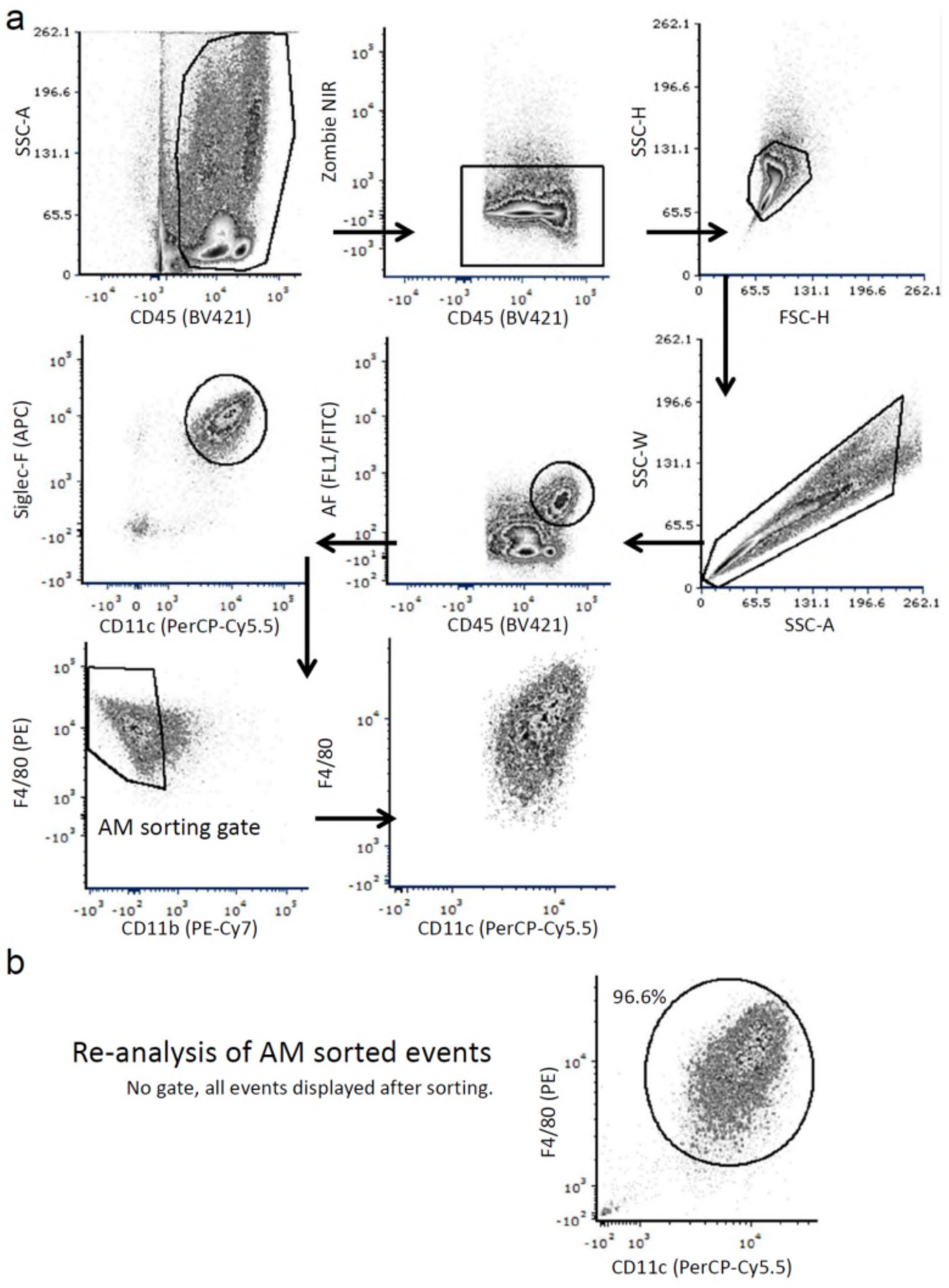

Supplementary Figure 9. Example gating strategy for alveolar macrophages sort. (a) After pre-gating $CD45^+$ leucocytes, dead cells were excluded by positive Zombie NIR staining. After debris and doublet exclusion, alveolar macrophages were defined as auto fluorescent (AF) positive, Siglec-F^+ $CD11c^+$ $CD11b^-$ cells. Their percentage of $CD45^+$ cells is displayed within Figure 4m. (b) Purity was confirmed by re-analysis of all events displayed within a $F4/80^+$ $CD11c^+$ dot plot (ungated).

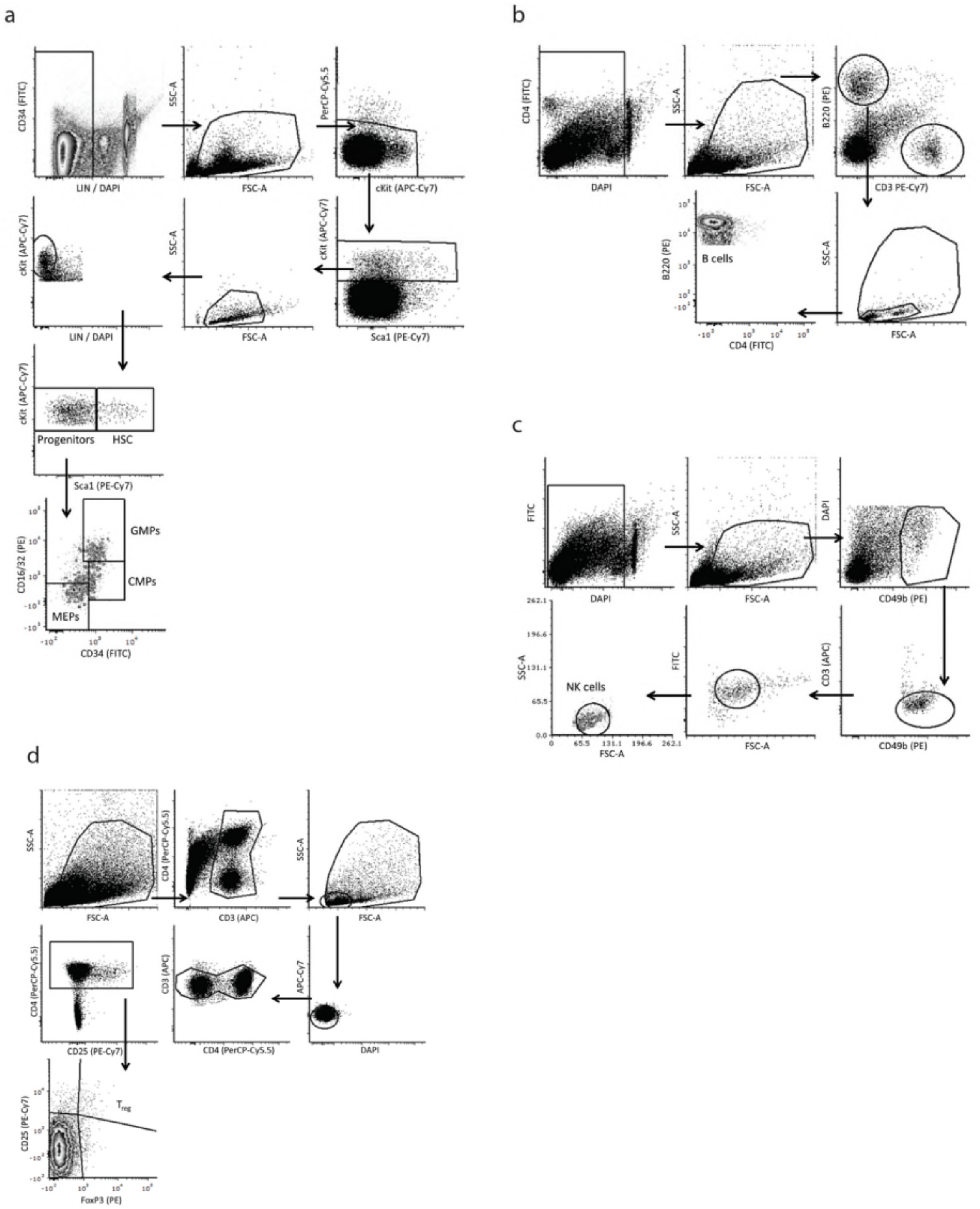

Supplementary 10. Gating strategies used in Figure 4. (a) Example gating strategy for hematopoietic stem cells (HSC), Progenitors, common myeloid progenitors (CMPs), granulocyte-monocyte progenitors (GMPs), and megakaryocyte-erythrocyte progenitors (MEPs) from bone marrow. Dead cells were excluded by positive DAPI staining together with LIN^+ cells. After diverse debris and doublet exclusions (using an empty channel (PerCP-Cy5.5) on the flow cytometer in addition) to purify the analyzed cells, the type of cells were defined as following: HSC (Lin^- $DAPI^-$ Sca^+ $cKit^+$), Progenitors (Lin^- $DAPI^-$ Sca^{low} $cKit^+$), CMPs (Lin^- $DAPI^-$ Sca^{low} $cKit^+$ $CD16/32^{low}$ $CD34^+$), GMPs (Lin^- $DAPI^-$ Sca^{low} $cKit^+$ $CD16/32^+$ $CD34^+$), and MEPs (Lin^- $DAPI^-$ Sca^{low} $cKit^+$ $CD16/32^-$ $CD34^-$). Their percentage of Lin^- $DAPI^-$ (within the FSC/SSC gate) were calculated and displayed within Figure 4b-f. (b) Example gating strategy for B cells (lung). Dead cells were excluded by positive DAPI staining. After defining a leukocyte gate (FSC/SSC, alive leukocytes), B cells were separated from T cells using B220 vs. CD3. Gated $B220^+$ cells were excluded from debris using a second smaller FSC/SSC gate. B cells were defined as $B220^+$ cells. B

cells were calculated as percentage of alive leukocytes (DAPI^{-} cells) and displayed within Figure 4g+j. Identical gating strategy was used analyzing spleen B cells. (c) Example gating strategy for NK cells (lung). Dead cells were excluded by positive DAPI staining. After defining a leukocyte gate (FSC/SSC, alive leukocytes), NK cells were separated from T cells using CD49b vs. CD3. After diverse debris and doublet exclusions (using an empty channel (FITC) on the flow cytometer in addition) to purify the analyzed cells, NK cells were defined as CD49b^{+} cells. NK cells were calculated as percentage of alive leukocytes (DAPI^{-} cells) and displayed within Figure 4h+l. Identical gating strategy was used analyzing spleen NK cells. (d) Example gating strategy for T_{Reg} cells (lung). T cells were separated by background debris and other cell types using a CD3^{+} gate. CD3^{+} cells were further separated by events using a lymphocyte gate (FSC/SSC, small gate). Using empty channels (DAPI and APC-Cy7) on the flow cytometer in addition, doublets and debris were excluded. T_{Reg} cells were defined as CD3^{+} CD4^{+} CD25^{+} FoxP3^{+} cells. T_{Reg} cells were calculated as percentage of CD3^{+} cells and displayed within Figure 4i+k. Identical gating strategy was used analyzing spleen T_{Reg} cells.

Supplementary Table 1. Humane endpoints in mice

Criteria	Observation	Score
**Body weight**	Unaffected (compared to PBS mean when considered pregnant)	0
	Reduction ≥ 10% (compared to PBS mean when considered pregnant)	5
	Reduction ≥ 15% (compared to PBS mean when considered pregnant)	10
	Reduction ≥ 20% (compared to PBS mean when considered pregnant)	15
	Reduction ≥ 25% (compared to PBS mean when considered pregnant)	20
**General condition**	Smooth fur, clean body openings	0
	Dull fur, cloudy eyes	5
	Mattened body openings, unnormal posture, high muscle tone, dehydration	10
	Cramps, paralysis	20
**Spontaneous behaviour**	Normal behaviour (sleep, reaction to contact, curiosity, social contacts)	0
	Unnormal behaviour, limited motor activity	5
	Isolation, pain utterance, apathic behaviour, significant hyper kinetics / stereotypic behaviour, coordination disorders	3,
	Automutilation	20
**Rating, measures**	No stress	0
	Weak stress: daily monitoring	5-9
	Moderate stress: monitoring twice a day if appropriate	≥ 10-19
	Moderate stress for more than 72 h is equal strong stress	20
	Strong stress: immediate euthanization	≥ 20

Supplementary Table 2. Reagents and Sources

REAGENT or RESOURCE	SOURCE	IDENTIFIER
Antibodies		
Influenza A Virus Nucleoprotein antibody [C43]	Abcam	RRID:AB_11143769
Anti-Mouse IgG (whole molecule)-Peroxidase antibody produced in goat	Sigma-Aldrich	RRID:AB_258167
Anti-Mouse CD11b (Integrin alpha M, Mac-1 alpha) Monoclonal Antibody, Alexa Fluor 647 Conjugated, [M1/70]	Thermo Fisher Scientific	RRID:AB_469780
Anti-Mouse CD11c (Integrin aX, p150 / 90) Monoclonal Antibody, Phycoerythrin-Cy5.5 (PE-Cy5.5) Conjugated, [N418]	Thermo Fisher Scientific	RRID:AB_469708
F4/80 Monoclonal Antibody [BM8], PE, eBioscience™	Thermo Fisher Scientific	RRID:AB_465923
Brilliant Violet 421™ anti-mouse CD45 antibody [30-F11]	BioLegend	RRID:AB_2562559
CD170 (Siglec F) Monoclonal Antibody [1RNM44N], eBioscience	Thermo Fisher Scientific	RRID:AB_2572866
CD45R (B220) Monoclonal Antibody [RA3-6B2], PE, eBioscience	Thermo Fisher Scientific	RRID:AB_465672
CD11b Monoclonal Antibody [M1/70], PE-Cyanine7, eBioscience	Thermo Fisher Scientific	RRID:AB_469588
CD11c Monoclonal Antibody [N418], PerCP-Cyanine5.5, eBioscience	Thermo Fisher Scientific	RRID:AB_925727
PE anti-mouse CD16/32 antibody [93]	BioLegend	RRID:AB_312807
CD25 Monoclonal Antibody [PC61.5], PE-Cyanine7, eBioscience	Thermo Fisher Scientific	RRID:AB_469608
APC/Cyanine7 anti-mouse CD3 antibody [145c11]	BioLegend	RRID:AB_2242784
CD3e Monoclonal Antibody [145-2C11], PE-Cyanine7, eBioscience	Thermo Fisher Scientific	RRID:AB_469571
CD34 Monoclonal Antibody [RAM34], FITC, eBioscience	Thermo Fisher Scientific	RRID:AB_465022
CD4 Monoclonal Antibody [RM4-5], FITC, eBioscience	Thermo Fisher Scientific	RRID:AB_464897
CD4 Monoclonal Antibody [RM4-59], PerCP-Cyanine5.5, eBioscience	Thermo Fisher Scientific	RRID:AB_1107001
CD49b (Integrin alpha 2) Monoclonal Antibody [DX5], PE, eBioscience	Thermo Fisher Scientific	RRID:AB_466072
APC/Cyanine7 anti-mouse CD117 (c-kit) antibody [2B8]	BioLegend	RRID:AB_1626278
FOXP3 Monoclonal Antibody [150D/E4], PE, eBioscience	Thermo Fisher Scientific	RRID:AB_10670338
PE/Cy7 anti-mouse Ly-6A/E (Sca-1) antibody [E13-161.7]	BioLegend	RRID:AB_756199
V450 Mouse Lineage Antibody Cocktail, BD Horizon	BD Bioscience	RRID:AB_10611731
APC Rat Anti-Mouse CD8a [53-6.7]	BD Bioscience	RRID:AB_10563416
CD86 (B7-2) Monoclonal Antibody [GL1], APC, eBioscience	Thermo Fisher Scientific	RRID:AB_469419
PE Rat Anti-Mouse Ly-6G [1A8]	BD Bioscience	RRID:AB_394208
Ly-6C Rat anti-Mouse, PerCP-Cyanine5.5, [HK1.4], eBioscience	Thermo Fisher Scientific	
MHC Class II (I-A/I-E) Monoclonal Antibody (M5/114.15.2), FITC, eBioscience	Thermo Fisher Scientific	RRID:AB_465232
PerCP/Cyanine5.5 anti-mouse CD206 (MMR) antibody [C068C2]	BioLegend	RRID:AB_2561992
Bacterial and Virus Strains		

Methicillin-resistant *Staphylococcus aureus* (USA300)	Martin Aepfelbacher, Medical Microbiology, Virology and Hygiene, University Medical Center Hamburg-Eppendorf, Hamburg, Germany	
A/Hamburg/NY1580/09 (H1N1) (2009 pH1N1)	Sigrid Baumgarte, Institut für Hygiene und Umwelt, Hamburg, Germany	
B/Lee/40	Thorsten Wolff, Robert Koch Institute, Berlin, Germany	
A/Aichi/63 (H3N2)<br>(6+2 gene reassortant in WSN background)	Eva Friebertshäuser-Böttcher, Institute for Virology, Philipps University Marburg	
Biological Samples		
Chemicals, Peptides, and Recombinant Proteins		
Poly(I:C); Polyinosinic-polycytidylic acid sodium salt, TLR receptor tested	Sigma Aldrich	Cat.: P9582-50MG
Collagenase D from Clostridium histolyticum	Sigma Aldrich	Cat.: 11088866001
DNase from bovine pancreas	Sigma Aldrich	Cat.: 11284932001
Critical Commercial Assays		
Procartaplex Cytokine Multiplex Assay<br>PPX-07: Mouse 7plex:MCP-1,IL-1 beta,IL-2,IL-6,IL-10,IL-17A (CTLA-8),TNF	Life Technologies	Assay-ID:MXGZFU3
Progesterone ELISA Kit	Cayman Chemical	RRID:AB_2811273
Corticosterone Enzyme Immunoassay Kit	Arbor Assays	Cat.: K014-H1
Deposited Data		
Experimental Models: Cell Lines		
Mardin-Darby Canine Kidney (MDCK II)	ATCC	RRID:CVCL_0422
Experimental Models: Organisms/Strains		
Mouse: C57BL/6J	Charles River	Strain Code 632
Mouse: Balb/cJRj	Janvier Labs	

Oligonucleotides		
Primer for sex determination SX_F 5´-GATGATTTGAGTGGAAATGTGAGGTA-3’		
Primer for sex determination SX_R 5´-CTTATGTTTATAGGCATGCACCATGTA-3’		
Random nonamere primer pd(N)9	Gene Link	Cat.: 26-4000-06
See supplementary table 2 for nutrient gene primers		
IBV NP primer FWD: 5’-GAACCCAGGGATTGCAGACA-3’		
IBV NP primer REV: 5’-ATGGGAAGCACCACTTTGCT-3’		
Recombinant DNA		
Software and Algorithms		
GraphPad Prism v.8.4.2	GraphPad Software Inc.	https://www.graphpad.com/scientific-software/prism/
BD FACS Diva Software v.8.0.1	BD Biosciences	https://www.bdbiosciences.com/eu/instruments/research/software/flow-cytometry-acquisition/bd-facsdiva-software/m/111112/features
ADVIA Centaur XP	Siemens Healthcare Diagnostics	https://www.siemens-healthineers.com/immunoassay/systems/advia-centaur-xp
flexiWare Software 7.6	Flexivent	https://www.scireq.com/2015/09/29/new-flexiware-76-software/
FCS Express 6	De Novo Software	https://denovosoftware.com/
Other		

Supplementary Table 2. Primers.

gene	forward 5'-3'	reverse 3' – 5'
Ywhaz	CACGCTCCCTAACCTTGCTT	ATCGTAGAAGCCTGACGTGG
Grb10	AAGCGAAGACCGAGATGAAG	CATAGGTGCGTTGAAAGGAG
Igf2	CTTGGATCCCAGAACCCAAGAA	CCCCTTGGTGACATGGGGAC
Sc36a1	CGGGAGAGTAGGAGGAGTCT	GTCTGCTCCCACACATCGTT
Slc38a2	AATGCGATTGTGGGCAGTGG	AGCTTTCCAGCCAGACCATAC
Sly/Xlr	GATGATTTGAGTGGAAATGTGAGGTA	CTTATGTTTATAGGCATGCACCATGTA
IBV *NP*	GAACCCAGGGATTGCAGACA	ATGGGAAGCACCACTTTGCT
