# Supplementary material for: Offspring born to influenza A virus infected pregnant mice have increased susceptibility to viral and bacterial infections in early life
Source: Nat Commun. 2021 Aug 16;12:4957. doi: 10.1038/s41467-021-25220-3 (PMC8368105; doi:10.1038/s41467-021-25220-3)
Supplement: Supplementary file 3 — Reporting Summary [file 41467_2021_25220_MOESM3_ESM.pdf]

nature research

Corresponding author(s): Gülsah Gabriel

Last updated by author(s): May 17, 2021

Reporting Summary

Nature Research wishes to improve the reproducibility of the work that we publish. This form provides structure for consistency and transparency in reporting. For further information on Nature Research policies, see our Editorial Policies and the Editorial Policy Checklist.

Statistics

For all statistical analyses, confirm that the following items are present in the figure legend, table legend, main text, or Methods section.

n/a	Confirmed	
☐	☒	The exact sample size (*n*) for each experimental group/condition, given as a discrete number and unit of measurement
☐	☒	A statement on whether measurements were taken from distinct samples or whether the same sample was measured repeatedly
☐	☒	The statistical test(s) used AND whether they are one- or two-sided<br>*Only common tests should be described solely by name; describe more complex techniques in the Methods section.*
☐	☒	A description of all covariates tested
☐	☒	A description of any assumptions or corrections, such as tests of normality and adjustment for multiple comparisons
☐	☒	A full description of the statistical parameters including central tendency (e.g. means) or other basic estimates (e.g. regression coefficient) AND variation (e.g. standard deviation) or associated estimates of uncertainty (e.g. confidence intervals)
☐	☒	For null hypothesis testing, the test statistic (e.g. *F*, *t*, *r*) with confidence intervals, effect sizes, degrees of freedom and *P* value noted<br>*Give P values as exact values whenever suitable.*
☒	☐	For Bayesian analysis, information on the choice of priors and Markov chain Monte Carlo settings
☒	☐	For hierarchical and complex designs, identification of the appropriate level for tests and full reporting of outcomes
☒	☐	Estimates of effect sizes (e.g. Cohen's *d*, Pearson's *r*), indicating how they were calculated

Our web collection on statistics for biologists contains articles on many of the points above.

Software and code

Policy information about availability of computer code

Data collection	BD FACS DivaTM Software v.8.0.1, ADVIA Centaur XP, flexiWare Software 7.6, Primer-BLAST (http://www.ncbi.nlm.nih.gov/tools/primer-blast/index.cgi)
Data analysis	GraphPad Prism v8.4.2, FCS Express 6

For manuscripts utilizing custom algorithms or software that are central to the research but not yet described in published literature, software must be made available to editors and reviewers. We strongly encourage code deposition in a community repository (e.g. GitHub). See the Nature Research guidelines for submitting code & software for further information.

Data

Policy information about availability of data

All manuscripts must include a data availability statement. This statement should provide the following information, where applicable:

- Accession codes, unique identifiers, or web links for publicly available datasets
- A list of figures that have associated raw data
- A description of any restrictions on data availability

All relevant data are available within the paper or its Supplementary Information files. Source data are provided with this paper.

Field-specific reporting

Life sciences study design

All studies must disclose on these points even when the disclosure is negative.

Sample size	No sample-size was pre-determined due to the explorative character of this study. Group sizes of this study were based on the study-specific animal project license. Therefore, group sizes reflect ethical and biometric considerations to allow for experiments providing sufficient data for explorative analysis. Animal experiments were designed to provide a minimal group size of (n = 3) for each condition tested. If animal experiments were repeated, for example maternal infection to generate offspring born to these animals, data collected from mothers were merged. This also applies to all data regarding pregnancy outcome and fetal / offspring's health, since more cohorts of offspring were bred for different downstream experiments. For offspring experiments, such as FACS-analysis, we aimed for a group size of (n = 4) with two offspring from two different mothers each to also respect maternal effects in group composition. Experiments were group building failed (because not enough offspring per sex was provided by breeding) were repeated and group size was set to (n = 8). This applies to the FACS measurement of alveolar macrophages in the offspring. Whenever sex-differences were statistically significant the data in this manuscript are presented in a sex-stratified manner. If sex was not identified as a confounding factor, data for this figure were merged and displayed without sex-stratification. This decision is always stated in the respective part of the manuscript.
Data exclusions	For Cytokine measurements and FACS analyses, technical and mathematical outliers were detected and excluded. The detection of technical outliers in cytokine measurements was based on evaluation of technical triplicate measurements. Mathematical outliers were defined using Grubb's test, when applicable. The detection of technical outliers during flow cytometry was based on technical problems during the experiment (usually, not enough cells were aquired).
Replication	Maternal infection experiments (Fig. 1, Sup.Fig. 1) were performed at least in two independent animal experiments and data were merged. Measurements of cytokines and hormones were performed utlizing technical triplicates. Cytokine measurements from the blood plasma of these mice was only possible once, since no more unthawed plasma was available at late stages of the study. These measurements cannot be repeated.<br><br>Experiments regarding gestational outcome (Fig. 2). were conducted in at least two indepentend experiments. Usually, all experiments that were necessary to generate offspring for downstream experiments were included to further strenghten the data.<br><br>Second hit experiments (Fig. 3, Sup. Fig. 4) for MRSA were performed in two independent experiments. Second hit experiments with IBV were only performed once, since one infection experiment failed (dose too low) and no additional animals were available on the animal experimental license. All individual experiments consisted of groups with at least two mothers per condition to respect maternal effects in group building. Cytokine measurements were performed in technical triplicates. Bacterial titration was performed in technical duplicates.<br><br>Flow cytometric analyses (Fig. 4, Sup. Fig. 6, 8 and 9) were performed once with four independend animals per group and sex (biologica replicates). As indicated above each group was recruited from two independend litters.<br><br>One adoptive transfer experiment (Fig. 5, Sup. Fig. 7) was performed with at least 5 sentinel animals (biological replicates) per group (depending on litter size and sex-distribution). Again, all groups were build from at least two litters. Donor animals were recruited the same way and macrophages were merged before transfer. Virus titration was performed in technical duplicates.<br><br>Assessment of lung function (Sup. Fig. 2 and 5) was performed once per age group with a biological replicate of (n = 8-9).<br><br>Homologous offspring challenge (Sup. Fig. 3) was performed once.<br><br>All attempts at replication were successful.
Randomization	For maternal infection experiments, no randomization was performed as all animals are inbreed and housed the same way. For composing offspring groups for downstream experiments, animals were assigned randomly by sex by animal caretakers prior to the experiment (random ID-assignment). Experimental groups were allocated in an age- and sex-dependent fashion as described in detail in the respective figure.
Blinding	No blinding was performed when using infectious animals due to biosafety regulations. Blinding was performed when handling and assessing non-infectious material.

Reporting for specific materials, systems and methods

We require information from authors about some types of materials, experimental systems and methods used in many studies. Here, indicate whether each material, system or method listed is relevant to your study. If you are not sure if a list item applies to your research, read the appropriate section before selecting a response.

Materials & experimental systems

n/a	Involved in the study	
	x	Antibodies
	x	Eukaryotic cell lines
x		Palaeontology and archaeology
	x	Animals and other organisms
x		Human research participants
x		Clinical data
x		Dual use research of concern

Methods

n/a	Involved in the study	
x		ChIP-seq
	x	Flow cytometry
x		MRI-based neuroimaging

Antibodies

Antibodies used

Influenza A Virus Nucleoprotein antibody [C43] Abcam RRID:AB_11143769 ab128193, dilution: 1:1000
Anti-Mouse IgG (whole molecule)-Peroxidase antibody produced in goat Sigma-Aldrich RRID:AB_258167 A4416, , dilution: 1:1000
Anti-Mouse CD11b (Integrin alpha M, Mac-1 alpha) Monoclonal Antibody, Alexa Fluor 647 Conjugated, [M1/70] Thermo Fisher Scientific RRID:AB_469780 19-0112-83, dilution: 1:100
Anti-Mouse CD11c (Integrin aX, p150 / 90) Monoclonal Antibody, Phycoerythrin-Cy5.5 (PE-Cy5.5) Conjugated, [N418] Thermo Fisher Scientific RRID:AB_469708 35-0114-81, dilution: 1:100
F4/80 Monoclonal Antibody [BM8], PE, eBioscience™ Thermo Fisher Scientific RRID:AB_465923 12-4801-82, , dilution: 1:100
Brilliant Violet 421™ anti-mouse CD45 antibody [30-F11] BioLegend RRID:AB_2562559 103134, dilution: 1:100
CD170 (Siglec F) Monoclonal Antibody [1RNM44N], eBioscience™ Thermo Fisher Scientific RRID:AB_2572866 14-1702-82 , dilution: 1:100
CD45R (B220) Monoclonal Antibody [RA3-6B2], PE, eBioscience™ Thermo Fisher Scientific RRID:AB_465672 12-0452-83, dilution: 1:100
CD11b Monoclonal Antibody [M1/70], PE-Cyanine7, eBioscience™ Thermo Fisher Scientific RRID:AB_469588 25-0112-82, dilution: 1:100
CD11c Monoclonal Antibody [N418], PerCP-Cyanine5.5, eBioscience™ Thermo Fisher Scientific RRID:AB_925727 45-0114-82, dilution: 1:100
PE anti-mouse CD16/32 antibody [93] BioLegend RRID:AB_312807 101308, dilution: 1:50
CD25 Monoclonal Antibody [PC61.5], PE-Cyanine7, eBioscience™ Thermo Fisher Scientific RRID:AB_469608 25-0251-82, dilution: 1:50
APC/Cyanine7 anti-mouse CD3 antibody [145c11] BioLegend RRID:AB_2242784 100222, dilution: 1:100
CD3e Monoclonal Antibody [145-2C11], PE-Cyanine7, eBioscience™ Thermo Fisher Scientific RRID:AB_469571 25-0031-81, dilution: 1:100
CD34 Monoclonal Antibody [RAM34], FITC, eBioscience™ Thermo Fisher Scientific RRID:AB_465022 11-0341-85, dilution: 1:50
CD4 Monoclonal Antibody [RM4-5], FITC, eBioscience™ Thermo Fisher Scientific RRID:AB_464897 11-0042-85, dilution: 1:100
CD4 Monoclonal Antibody [RM4-59], PerCP-Cyanine5.5, eBioscience™ Thermo Fisher Scientific RRID:AB_1107001 45-0042-82, dilution: 1:100
CD49b (Integrin alpha 2) Monoclonal Antibody [DX5], PE, eBioscience™ Thermo Fisher Scientific RRID:AB_466072 12-5971-81, dilution: 1:100
APC/Cyanine7 anti-mouse CD117 (c-kit) antibody [2B8] BioLegend RRID:AB_1626278 105826, dilution: 1:50
FOXP3 Monoclonal Antibody [150D/E4], PE, eBioscience™ Thermo Fisher Scientific RRID:AB_10670338 12-4774-42, dilution: 1:50
PE/Cy7 anti-mouse Ly-6A/E (Sca-1) antibody RRID: AB_469669, dilution: :50
V450 Mouse Lineage Antibody Cocktail, BD Horizon BD Bioscience RRID:AB_10611731 561301, dilution: 1:5
APC Rat Anti-Mouse CD8a [53-6.7] BD Bioscience RRID:AB_10563416 561093, dilution: 1:100
CD86 (B7-2) Monoclonal Antibody [GL1], APC, eBioscience™ Thermo Fisher Scientific RRID:AB_469419 17-0862-82, dilution: 1:50
PE Rat Anti-Mouse Ly-6G [1A8] BD Bioscience RRID:AB_394208 551461, dilution: 1:100
Ly-6C Rat anti-Mouse, PerCP-Cyanine5.5, [HK1.4], eBioscience™ Thermo Fisher Scientific RRID: AB_2723343 45-5932-82, dilution: 1:100
MHC Class II (I-A/I-E) Monoclonal Antibody (M5/114.15.2), FITC, eBioscience™ Thermo Fisher Scientific RRID:AB_465232 11-5321-82 , dilution: 1:50
PerCP/Cyanine5.5 anti-mouse CD206 (MMR) antibody [C068C2] BioLegend RRID:AB_2561992 141716, dilution: 1:50
Biotin-SP (long spacer) AffiniPure F(ab')$_2$ Fragment Donkey Anti-Rabbit IgG (H+L), RRID: AB_2340594, 711-066-152, dilution: 1:10000

Validation

Primer antibodies were validated by including appropriate controls as described in detail in the manuscript.

Eukaryotic cell lines

Policy information about cell lines

Cell line source(s)

Mardin-Darby Canine Kidney (MDCK II) ATCC RRID:CVCL_0422

Authentication

No authentication was performed after receiving the cells from the supplier.

Mycoplasma contamination	All cells were tested negative for Myoplasma.
Commonly misidentified lines (See ICLAC register)	No commonly misidentified cell lines were used.

Animals and other organisms

Policy information about studies involving animals; ARRIVE guidelines recommended for reporting animal research

Laboratory animals	Mouse (Mus musculus), C57Bl/6J, female, 8 – 10 week old at experiment start<br>Mouse (Mus musculus), BALB/c, male, 8 – 12 week old for breeding<br>Mouse (Mus musculus), C57/Bl6J+BALB/c, male and female, 0 – 24 week old, depending on experiment and readout, as indicated in the manuscript
Wild animals	No wild animals were used in this study.
Field-collected samples	No field collected samples were used in this study.
Ethics oversight	All animal experiments were performed according to the guidelines of animal protection law and the approved protocols by the relevant German authority (Behörde für Gesundheit und Verbraucherschutz Hamburg, approval number 124/12, 75/17, 97/19 and Behörde für Gesundheit, Verbraucherschutz und Pharmazie, Regierung von Oberbayern, approval number 46-16).

Note that full information on the approval of the study protocol must also be provided in the manuscript.

Flow Cytometry

Plots

Confirm that:

- [x] The axis labels state the marker and fluorochrome used (e.g. CD4-FITC).
- [x] The axis scales are clearly visible. Include numbers along axes only for bottom left plot of group (a 'group' is an analysis of identical markers).
- [ ] All plots are contour plots with outliers or pseudocolor plots.
- [ ] A numerical value for number of cells or percentage (with statistics) is provided.

Methodology

Sample preparation	Lung tissue was minced on ice and incubated in lysis buffer (2.5 mg/ml Collagenase D, 0.01 mg/ml DNase I in RPMI) at 37 °C for 30 min with constant agitation. Bone marrow was isolated by centrifugation from murine tibia and femur at 1000 g for 5 min at 4 °C. Single cell solutions were prepared using a 70 µm cell strainer (Corning) in FACS buffer (2% FCS in PBS). Red blood cells were lysed using RBC lysis buffer (BioLegend) for 5 min on ice. Life/dead staining was performed using ZombieTM Nir Fixable Viability Kit (BioLegend) for alveolar macrophages or DAPI for other populations following the manufacturer's instructions. Antibody staining was performed for 30 min at RT in the dark with an antibody concentration of 6 µl/ml. For antibody details see key resource table. Stained cells were resuspended in FACS buffer containing 2% BSA for subsequent analysis or sorts. Flow cytometric analysis as well as FACS was performed using a FACSAria II Fusion SORP (BD), analysis was performed on a FACSCantoII and data analysis was done using FACSDiva software v 8.0 as well as FCS Express 6 (DeNovo Software, USA). A representative example gating strategy that was used for sorting AMs and the confirmed purity of the sort (re-analysis) is shown in the Supplementary Figures 9 and 10.<br>Cll population abundance:<br>Post-sort fractions contained >95.6% CD11c+ F4/80+ events (determined as alveolar macrophages) of all events recorded (without prior gating); one representative example of the re-analysis is visualized within Suppl. Fig 10, b.
Instrument	FACS Aria-Fusion (5-laser, 18-fluorescence system)
Software	BD FACS DivaTM Software v.8.0.1
Cell population abundance	*Describe the abundance of the relevant cell populations within post-sort fractions, providing details on the purity of the samples and how it was determined.*
Gating strategy	*Describe the gating strategy used for all relevant experiments, specifying the preliminary FSC/SSC gates of the starting cell population, indicating where boundaries between "positive" and "negative" staining cell populations are defined.*

- [x] Tick this box to confirm that a figure exemplifying the gating strategy is provided in the Supplementary Information.
